# Whole exome sequencing in neurogenetic odysseys: An effective, cost- and time-saving diagnostic approach

**DOI:** 10.1371/journal.pone.0191228

**Published:** 2018-02-01

**Authors:** Marta Córdoba, Sergio Alejandro Rodriguez-Quiroga, Patricia Analía Vega, Valeria Salinas, Josefina Perez-Maturo, Hernán Amartino, Cecilia Vásquez-Dusefante, Nancy Medina, Dolores González-Morón, Marcelo Andrés Kauffman

**Affiliations:** 1 Consultorio de Neurogenética, Centro Universitario de Neurologia y Division Neurologia, Hospital J.M.Ramos Mejia, Facultad de Medicina, UBA, Buenos Aires, Argentina; 2 Programa de Medicina de Precisión y Genómica Clínica, Instituto de Investigaciones en Medicina Traslacional, Facultad de Ciencias Biomédicas, Universidad Austral-CONICET, Buenos Aires, Argentina; 3 Servicio de Neurología Infantil, Hospital Universitario Austral, Buenos Aires, Argentina; Charite Universitatsmedizin Berlin, GERMANY

## Abstract

**Background:**

Diagnostic trajectories for neurogenetic disorders frequently require the use of considerable time and resources, exposing patients and families to so-called “diagnostic odysseys”. Previous studies have provided strong evidence for increased diagnostic and clinical utility of whole-exome sequencing in medical genetics. However, specific reports assessing its utility in a setting such as ours- a *neurogeneticist led* academic group serving in a low-income country—are rare.

**Objectives:**

To assess the diagnostic yield of WES in patients suspected of having a neurogenetic condition and explore the cost-effectiveness of its implementation in a research group located in an Argentinean public hospital.

**Methods:**

This is a prospective study of the clinical utility of WES in a series of 40 consecutive patients selected from a Neurogenetic Clinic of a tertiary Hospital in Argentina. We evaluated patients retrospectively for previous diagnostic trajectories. Diagnostic yield, clinical impact on management and economic diagnostic burden were evaluated.

**Results:**

We demonstrated the clinical utility of Whole Exome Sequencing in our patient cohort, obtaining a diagnostic yield of 40% (95% CI, 24.8%-55.2%) among a diverse group of neurological disorders. The average age at the time of WES was 23 (range 3–70). The mean time elapsed from symptom onset to WES was 11 years (range 3–42). The mean cost of the diagnostic workup prior to WES was USD 1646 (USD 1439 to 1853), which is 60% higher than WES cost in our center.

**Conclusions:**

WES for neurogenetics proved to be an effective, cost- and time-saving approach for the molecular diagnosis of this heterogeneous and complex group of patients.

## Introduction

Neurogenetic disorders are a frequent reason for medical consultation in neurology services. Clinical variability and genetic heterogeneity are a hallmark of these diseases. Their diagnostic approach requires extensive clinical, radiological and genetic evaluations. Moreover, many of these procedures are invasive and costly. However, despite the use of considerable time and resources, the diagnostic yield in this field has been disappointingly low. This etiologic search has been called a “diagnostic odyssey” for many families [1].

Whole Exome Sequencing (WES) has proved to be a valuable tool in medical genetics, for diagnostic and gene discovery purposes [2–4]. Although a diagnostic yield of about 30% in neurogenetic disorders can be extrapolated from the results of large series that have included other medical conditions as well [5], specific reports assessing its utility in a setting such as ours—a *neurogeneticist led* academic group serving in a low-income country—are rare. Therefore, there is still a necessity to assess its clinical utility and the feasibility of its implementation for neurogenetic diagnostic practice in less economic favorable locations where rational and effective use of resources is both an obligation and an opportunity for reducing inequalities [6, 7].

We are reporting here on our first 40 consecutive cases which were selected from our research-based laboratory for WES. We demonstrated the clinical utility of WES and the potential cost-effectiveness of WES as a single test by examining the number and types of tests that were performed prior to WES that add to the cost of diagnostic workups.

## Materials and methods

### Clinical samples

We included a consecutive series of 40 patients selected for WES from a Neurogenetic Clinic of a tertiary Hospital in Argentina. These patients were considered candidates for genomic studies according to the presence of typical findings of known neurogenetic diseases and/or hints of monogenic etiology such as familial aggregation or chronic and progressive course. We recorded perinatal and family history, likely inheritance model/s, disease progression characteristics, comorbidities, and studies performed before WES from each patient of our cohort. The diverse clinical features of this cohort are summarized in Table 1. Written informed Consent for WES was obtained from the patients and/or their family. The informed consent included the option to receive or not incidental findings according to ACMG recommendations. Internal review board (IRB) approval was obtained at Hospital JM Ramos Mejia. All methods were performed in accordance with the relevant guidelines and regulations.

**Table 1:** Demographic characteristics and clinical features of patients selected for WES (*).

CASE ID	AGE OF ONSET	AGE AT TESTING	PRIMARY DISEASE CLASSIFICATION	CLINICAL PRESENTATION
1	1	28	-	Mental retardation, autism, epilepsy, dystonia
2	5	9	Epilepsy with Variable Foci	Epilepsy
3	1	5	Dravet Syndrome	Epilepsy, cognitive impairment
4	9	17	Hemiplegic Migraine	Episodic migraine, hemiplegia
5	14	24	Sporadic ataxia	Ataxia, myoclonus, cognitive impairment, cerebellar atrophy on MRI
6	9	24	Spastic Paraplegia Plus	Paraplegia, mental retardation, thinning of the corpus callosum on MRI, peripheral neuropathy
7	4	23	-	Generalized dystonia, chorea, cognitive impairment
8	2	5	Epileptic encephalopathy	Ataxia, absence epilepsy, neurodevelopmental delay
9	8	50	Myopathy	Very mild muscle weakness, hyperCKemia
10	1	11	Epileptic encephalopathy	Autism, hyperactivity, epilepsy
11	6	11	Ataxia + oculomotor apraxia	Ataxia, chorea, tremor, oculomotor apraxia
12	16	23	Leukodystrophy	leukodystrophy on MRIs + cognitive impairment Ataxia + pyramidal syndrome + abnormal eye movements
13	55	70	Sporadic ataxia	Ataxia
14	1	4	Leigh syndrome	Developmental delay, refractory epileptic encephalopathy, MRI signal abnormalities in the basal ganglia
15	11	22	Mitochondrial Disorder	Muscle fatigue
16	1	5	Chain respiratory disorder	Developmental delay, recurrent vomiting
17	29	54	Sporadic ataxia	Ataxia, pyramidal.
18	5	15	Ataxia	Ataxia, neuropathy, cerebellar atrophy
19	2	12	-	Developmental Disorder, speech impairment, polyneuropathy
20	42	53	Sporadic ataxia	Ataxia, cerebellar atrophy
21	3	11	Epileptic encephalopathy	Partial seizures, ataxia
22	Neonatal	3	Neonatal adrenoleukodystrophy	Hepatic dysfunction, hypotonia, white matter lesions on MRI
23	Neonatal	3	Encephalopathy	Mental delay, physical growth retardation, diarrhea, vomiting and increased lactic acid
24	Neonatal	9	Encephalopathy	Developmental delay, seizures, muscular weakness, dystonia. Fragmentary hypo myelination on MRI
25	30	52	Episodic ataxia	Episodic ataxia
26	12	23	Leukodystrophy	Ataxia, cognitive impairment, abnormal ocular movements. Symmetric hypo myelination on MRI
27	27	33	Rhabdomyolysis	Rhabdomyolysis, muscular fatigue
28	6m	5	Mitochondrial	Developmental delay, epilepsy, dystonia, ragged red fibers on muscular biopsy
29	3	32	Myopathy	Proximal muscular weakness, muscular atrophy
30	Neonatal	8	Congenital disorder of Glycosylation	Microcephaly, seizures, muscular weakness
31	Neonatal	10	Polymicrogyria	Seizures, polymicrogyria on MRI
32	2	8	-	Speech impairment, developmental delay
33	18m	31	Spastic quadriplegia	Quadriplegia, pyramidal dysfunction, fasciculation, muscular atrophy
34	50	58	Ataxia / Dementia	Progressive multidomain cognitive impairment, ataxia
35	6m	5	Myopathy	Developmental delay, hypotonia, muscular weakness
36	8	19	Dystonia	Generalized dystonia
37	2	16	Optic Neuropathy	Progressive visual loss
38	41	53	Sensory Ataxia	Ataxia, distal hypoesthesia
39	6	17	NBIA	Dystonia, tremor
40	46	56	Sub-acute Dementia-Movement Disorders	Behavioral disorders, tremor, bradykinesia

*36 patients were selected for WES based on the presence of a well-defined clinical syndrome; the first-tier analysis was done by investigating a panel of known disease genes known to be associated with the respective condition. The rest represents complex phenotypes with overlapping neurological features. The mean age at WES was 23, ranging from 3–70 years. (Age at testing column)

The mean time between the disease onset and WES was 11.5 years (range 3–42).

### Whole exome sequencing and sanger confirmation

Genomic DNA was isolated from blood samples of each subject with the use of commercial kits. DNA sequencing libraries were constructed mostly by chemical fragmentation using commercial preparation kits. Exomes were enriched using different systems, being the vast majority of our cases processed with SureSelect Human All Exon v4 Kits (Agilent Technologies, Santa Clara, CA, USA). NGS sequencing runs were made in Illumina HiSeq 2500 systems as an outsourced service from Macrogen Inc (Korea) obtaining an average sequence coverage of more than 70X, with more than 97% of the target bases having at least 10X coverage. All standardized procedures were performed according to manufacturer’s instructions that have been widely mentioned in the literature [8, 9]. Clinically relevant variants, from proband and parental samples (whenever available), were confirmed by Sanger sequencing.

### Data analysis and annotation

Sequence data in FastQ format were aligned to the human reference genome (GRCh37) using the Burrows-Wheeler Alignment Tool (BWA-MEM) [10]. Variants Calls were generated using GATK haplotype caller following the so called *best practices* [11]. The output vcf file was annotated at various levels using Annovar [12] (S1A Fig). Variants were prioritized according to inheritance model, population frequency, molecular function and effects of mutations, reported clinical effect, and optionally according to a list of genes associated with the disease under study. In that sense two in-house protocols were defined. One “molecular hypothesis free”, for patients presenting complex phenotypes without candidate genes. Another “molecular hypothesis targeted” for patients that shows a defined clinical syndrome with available candidate genes. (S1B Fig). Classification of variants followed previously published schemes [13] updated with recent recommendations and guidelines by the American College of Medical Genetics and Genomics and the Association for Molecular Pathology [14]. Joining variant level and clinical features information, we classified each WES study as **positive** if *a pathogenic/likely pathogenic mutation in known disease gene was identified with positive phenotypic and inheritance overlap*; **undetermined** if *a pathogenic/likely pathogenic mutation in a putative candidate gene was identified with positive phenotypic and inheritance overlap* or *only one pathogenic/likely pathogenic mutation was identified with positive phenotypic overlap in a recessive disorder* and **negative** *in the rest of the cases*. We paid special attention to reviews of previous work done in cases studied before the 2015 update, reanalyzing them according to the new schema. Details for each novel variant are presented in S1 Table.

Incidental findings were informed according to ACMG recommendations. Counseling to patients was performed by trained professionals.

## Results

**Fig 1:**
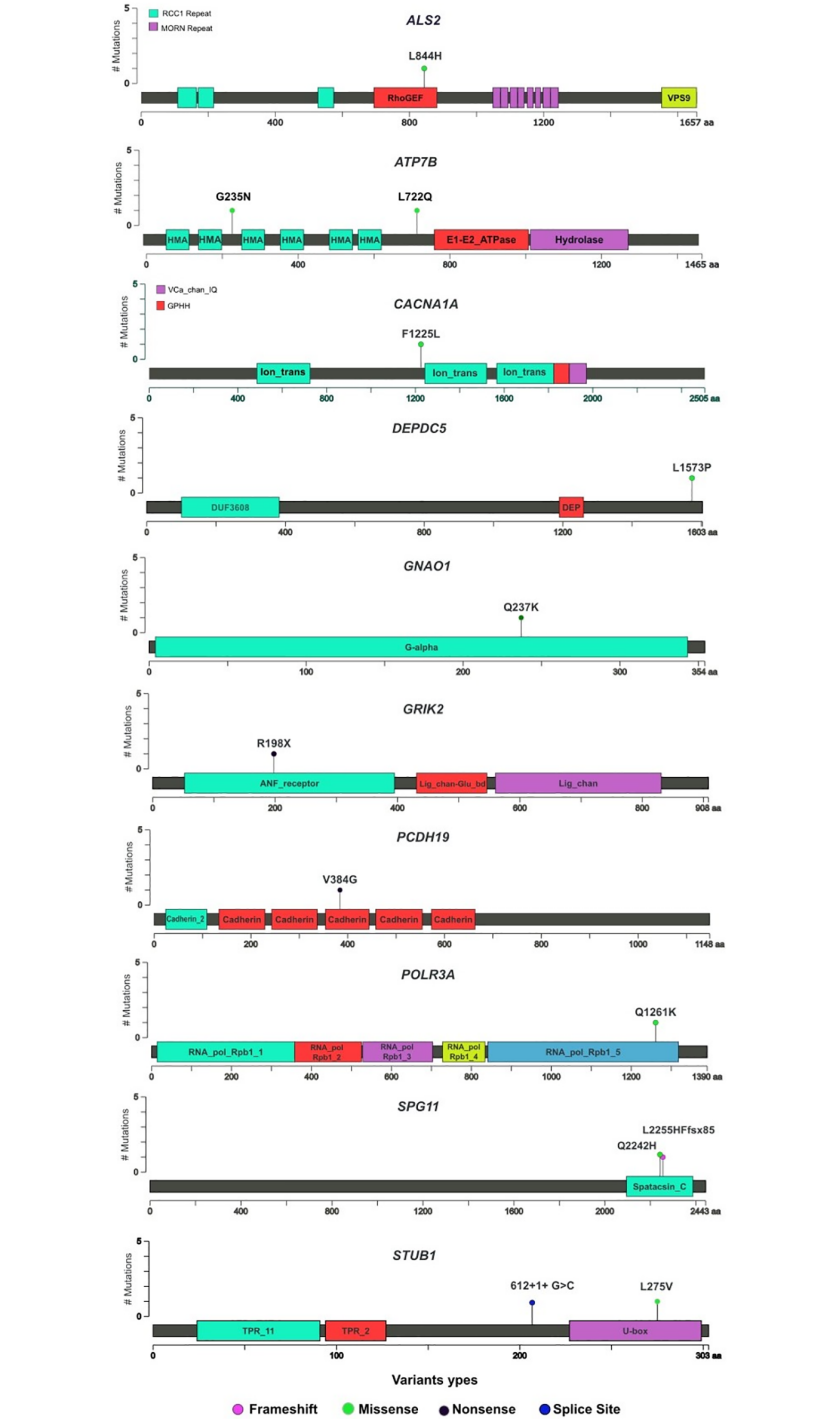
Location and impact of novel variants identified by this study.

WES proved to be an effective, cost- and time-saving diagnostic approach in our setting. Sixteen WES satisfied criteria for a full molecular diagnosis (Table 2 and S1 Table), thus the overall diagnostic yield for WES in our series was 40% (S2 Fig, Yield). Among them, two WES were reclassified from original undetermined and negative categories after subsequent reanalysis identified pathogenic variants in genes not associated with human disorders at the time of original reports. A diverse group of neurological disorders were represented in the positive patients (Table 2). The average age at the time of WES was 23 (3–70). The mean time elapsed from symptom onset to WES was 11 years (range 3–42). The positive group included 9 patients with autosomal dominant disease and 7 with autosomal recessive disease. Different mutation types were observed in this cohort. Noteworthy, 56% of the mutations were novel, according to ExAC v3 database [9] (Fig 1). Although almost all of the molecular diagnoses were in nuclear genes, mitochondrial genome sequencing included in the WES test yielded one diagnosis (one individual with a missense mutation in MT-T8993G.

**Table 2:** Summary of patients with established molecular diagnosis by WES.

**CASE ID**	**GENE**	**PHENOTYPE**	**OMIM Entry**	**INHERITANCE/ SEGREGATION**	**MUTATION(S)**	**LITERATURE**	**TYPE OF MUTATION**	**ALTERED MANAGEMENT**
1 (*)	*GRIK2*	Mental Retardation, autism, epilepsy, dystonia	611092	Recessive (Both parents inheritance)	NM_021956.4:c592C>T; p.R198X Hom	(Motazacker MM et al. 2007)	*nonsense*	
2	*DEPDC5*	Epilepsy with Variable Foci	604364	Dominant (paternal inheritance)	NM_001242896:c.4718T>C;p.L1573P	(Baulac et al. 2014)	*missense*	
4	*CACNA1A*	Hemiplegic Migraine	141500	Sporadic (De novo)	NM_000068:c.3675C>A; p.F1225L	(Riant et al. 2010)	*missense*	
5 (**)	*STUB1*	Sporadic Ataxia	607207	Sporadic (Both parents inheritance)	NM_005861.2:c.612+1 G> C; p.? NM_005861.2:c.823C>G;L275V	(Shi et al. 2014)	*splicing/missense*	Endocrine monitoring to evaluate appearance of hypogonadism
6	*SPG11*	Paraplegia, mental retardation, thinning of the corpus callosum peripheral neuropathy	604360	Sporadic (Both parents inheritance)	NM_025137:c.6763insA; p.L2255Hfsx85 NM_025137:6726A>T; p.Q2242H;	(Stevanin et al. 2007)	*Frameshift/ missense*	L-Dopa Trial
8	*KCNA2*	Ataxia, early absence epilepsy, neurodevelopmental delay	616366	Sporadic (De novo)	NM_001204269::c.G890A:p.R297Q (a)	(Syrbe et al. 2015)	*missense*	Acetazolamide and Fampridine Trial
9	*DMD*	Myopathy with very mild muscle weakness, hyperCKemia	300377	Sporadic	NM_004006.2:c.1149+1C>A (b) Het	(Carsana et al. 2010)	*splicing*	Avoid Statins
11	*APTX*	Ataxia, chorea, tremor, oculomotor apraxia	208920	Recessive (Both parents inheritance)	NM_175069.1:c.879G>A; p.W293X (c) Hom	(Shimazaki et al. 2002)	*nonsense*	Ubiquinone Trial
21	*PCDH19*	Epileptic encephalopathy with partial seizures and ataxia	300088	Sporadic (paternal inheritance)	NM_001184880:exon1:c.T1151G:p.V384G	(Hynes et al. 2010)	*nonsense*	
22	*PEX12*	Neonatal adrenoleukodystrophy with hepatic dysfunction, hypotonia, white matter lesions on MRI	266510	Sporadic (Both parents inheritance)	NM_000286:c.733_734insGCC;p.L245Cfsx19 (d) NM_000286:c.533_535del:p.Q178del (e)	(Gootjes et al. 2004)	*Frameshift/nonframeshift*	
26	*POLR3A*	Leukodystrophy with ataxia, cognitive impairment, abnormal ocular movements and symmetric hypo myelination on MRI	607694	Recessive (Both parents inheritance)	NM_007055.3:c.3781G>A; p.Q1261KNM_007055.3:c.3014G>A;p.R1005H (f)	(Wolf et al. 2014)	*Missense/missense*	
28	*MT-ATP6*	Mitochondrial disease with ddevelopmental delay, epilepsy, dystonia, ragged red fibers on muscular biopsy	551500	Mitochondrial	m.T8993G (g)	(Holt et al. 1990)	*missense*	Avoid drugs with mitochondrial toxicity
29	*SGCG*	Myopathy with proximal muscular weakness, muscular atrophy	608896	Sporadic (both parents inheritance)	NM_000231: c.521delT:p.F175LfsX20 (h) Hom	(Lasa et al. 1998)	*frameshift*	
30	*GNAO1*	Glycosylation congenital disorder with microcephaly, seizures, muscular weakness	615473	Sporadic (De novo)	NM_020988: c.709G>A:p.Q237K	(Nakamura et al. 2013)	*missense*	
33	*ALS2*	Spastic quadriplegia, pyramidal dysfunction, fasciculation, muscular atrophy	607225	Sporadic (both parents inheritance)	NM_020919: c.T2531A: p.L844H Hom	(Eymard-Pierre et al. 2006)	*missense*	
40 (***)	*ATP7B*	Sub-acute Dementia with movement Disorders	277900	Recessive (Both parents inheritance)	NM_000053: c.2165T>A: p.L722Q NM_000053: c.3704G>A: p.G235N	(Takeshita et al. 2002)	*Missense*/*missense*	Treatment with Penicilamine
**CASE ID**	**GENE**	**PHENOTYPE**	**OMIM Entry**	**INHERITANCE/ SEGREGATION**	**MUTATION(S)**	**LITERATURE**	**TYPE OF MUTATION**	**ALTERED MANAGEMENT**
1 (*)	*GRIK2*	Mental Retardation, autism, epilepsy, dystonia	611092	Recessive (Both parents inheritance)	NM_021956.4:c592C>T; p.R198X Hom	(Motazacker MM et al. 2007)	*nonsense*	
2	*DEPDC5*	Epilepsy with Variable Foci	604364	Dominant (paternal inheritance)	NM_001242896:c.4718T>C;p.L1573P	(Baulac et al. 2014)	*missense*	
4	*CACNA1A*	Hemiplegic Migraine	141500	Sporadic (De novo)	NM_000068:c.3675C>A; p.F1225L	(Riant et al. 2010)	*missense*	
5 (**)	*STUB1*	Sporadic Ataxia	607207	Sporadic (Both parents inheritance)	NM_005861.2:c.612+1 G> C; p.? NM_005861.2:c.823C>G;L275V	(Shi et al. 2014)	*splicing/missense*	Endocrine monitoring to evaluate appearance of hypogonadism
6	*SPG11*	Paraplegia, mental retardation, thinning of the corpus callosum peripheral neuropathy	604360	Sporadic (Both parents inheritance)	NM_025137:c.6763insA; p.L2255Hfsx85 NM_025137:6726A>T; p.Q2242H;	(Stevanin et al. 2007)	*Frameshift/ missense*	L-Dopa Trial
8	*KCNA2*	Ataxia, early absence epilepsy, neurodevelopmental delay	616366	Sporadic (De novo)	NM_001204269::c.G890A:p.R297Q (a)	(Syrbe et al. 2015)	*missense*	Acetazolamide and Fampridine Trial
9	*DMD*	Myopathy with very mild muscle weakness, hyperCKemia	300377	Sporadic	NM_004006.2:c.1149+1C>A (b) Het	(Carsana et al. 2010)	*splicing*	Avoid Statins
11	*APTX*	Ataxia, chorea, tremor, oculomotor apraxia	208920	Recessive (Both parents inheritance)	NM_175069.1:c.879G>A; p.W293X (c) Hom	(Shimazaki et al. 2002)	*nonsense*	Ubiquinone Trial
21	*PCDH19*	Epileptic encephalopathy with partial seizures and ataxia	300088	Sporadic (paternal inheritance)	NM_001184880:exon1:c.T1151G:p.V384G	(Hynes et al. 2010)	*nonsense*	
22	*PEX12*	Neonatal adrenoleukodystrophy with hepatic dysfunction, hypotonia, white matter lesions on MRI	266510	Sporadic (Both parents inheritance)	NM_000286:c.733_734insGCC;p.L245Cfsx19 (d) NM_000286:c.533_535del:p.Q178del (e)	(Gootjes et al. 2004)	*Frameshift/nonframeshift*	
26	*POLR3A*	Leukodystrophy with ataxia, cognitive impairment, abnormal ocular movements and symmetric hypo myelination on MRI	607694	Recessive (Both parents inheritance)	NM_007055.3:c.3781G>A; p.Q1261KNM_007055.3:c.3014G>A;p.R1005H (f)	(Wolf et al. 2014)	*Missense/missense*	
28	*MT-ATP6*	Mitochondrial disease with ddevelopmental delay, epilepsy, dystonia, ragged red fibers on muscular biopsy	551500	Mitochondrial	m.T8993G (g)	(Holt et al. 1990)	*missense*	Avoid drugs with mitochondrial toxicity
29	*SGCG*	Myopathy with proximal muscular weakness, muscular atrophy	608896	Sporadic (both parents inheritance)	NM_000231: c.521delT:p.F175LfsX20 (h) Hom	(Lasa et al. 1998)	*frameshift*	
30	*GNAO1*	Glycosylation congenital disorder with microcephaly, seizures, muscular weakness	615473	Sporadic (De novo)	NM_020988: c.709G>A:p.Q237K	(Nakamura et al. 2013)	*missense*	
33	*ALS2*	Spastic quadriplegia, pyramidal dysfunction, fasciculation, muscular atrophy	607225	Sporadic (both parents inheritance)	NM_020919: c.T2531A: p.L844H Hom	(Eymard-Pierre et al. 2006)	*missense*	
40 (***)	*ATP7B*	Sub-acute Dementia with movement Disorders	277900	Recessive (Both parents inheritance)	NM_000053: c.2165T>A: p.L722Q NM_000053: c.3704G>A: p.G235N	(Takeshita et al. 2002)	*Missense*/*missense*	Treatment with Penicilamine

Dominant inheritance was defined by the presence of an affected parent and recessive inheritance defined by unaffected parents and affected siblings

(a) ClinVar #190328; (b) UMD-DMD France Mutation Database Records 14050 and 18392; (c) ClinVar #4431; (d) and (e) cited in Mol Genet Metab. 2004 Nov;83(3):252–63; (f) ClinVar #31149; (g) ClinVar #9461; (h) ClinVar #2004;

(*) Further details were published in Clin Genet. 2015 Mar;87(3):293–5. doi: 10.1111/cge.12423.

(**) Further details were previously published in Neurology. 2014 Jul 15;83(3):287–8.

(***) Further details were previously published in Parkinsonism Relat Disord. 2015 Nov;21(11):1375–7.

As we mention in methods, a WES study is considered *positive* if pathogenic or likely pathogenic variants correspond to the phenotype and the mode of inheritance. We must recognize that only in case 33, this criterion is not strictly accomplished because the identified variant in *ALS2* must be considered of unknown significance according to last ACMG criteria. However, we discussed this situation with the referring physician and the patient’s family and decided to consider the *ALS2* variant likely causing the disease, despite acknowledging a higher uncertainty in diagnostic terms. According to this clinical decision, we included this case as a positive one into this work.

WES were defined as undetermined in two cases (5%). In one of them, we were able to identify only one pathogenic variant (NM_018082.5:c.1568T>A; p.V523Q) in *POLR3B* in a patient showing clinical features consistent with autosomal recessive *POLR3*-related disorders [15]. We hypothesize that the second missing allele is a large deletion/insertion or a deep intronic mutation. This case highlights current limitations of WES. In case 17, we found a heterozygous likely pathogenic variant (NM_030954.3:c.668C>A; p.A223N) in *RNF170* gene. This gene was reported as a cause of sensory ataxia [16]. The patient’s phenotype corresponds to pure cerebellar ataxia.

Table 2 shows a summary of the impact that a definitive diagnosis obtained from WES had on our patients. The information obtained by means of WES ended the diagnostic odysseys, led to therapeutic trials in some cases and improved genetic counselling processes with more precise information.

As an exploratory approach to a monetary cost-analysis of WES in neurogenetic diseases, we recorded the number and type of complementary tests done by our patients before WES. The average cost of the “expendable” diagnostic workup prior to WES was USD 1646 (USD 1439 to 1853), which is 60% higher than WES cost in our center (USD 1000). Table 3 shows that several genetic and non-genetic assays considered unnecessary (e.g. repetitive neuroimages and non-genetic assays) and/or evitable (e.g. recurrent outpatients visits and single-gene testing) were performed in almost all of our patients. This often-unnecessary repetition of complementary studies might be a consequence of the extension in time of the so-called *diagnostic odyssey* (see before results about time at WES since symptom onset). A more conservative analysis that added up WES cost and stratified the cohort into solved and unsolved cases showed differences too. The average cost of the diagnostic work up (including WES, expendable and non-expendable procedures) in solved cases was USD 4572 (USD 4302 to 4842), whereas in unsolved cases was USD 4514 (USD 4289 to 4739). Avoiding expendable procedures, by means of WES, could reduce diagnostic work up expenses in about 39% (USD 2792; 95% CI, USD 2634–2950).

**Table 3:** Summary of procedures* and visits* performed during the *Diagnostic Odysseys*.

Case id	CT	MRI	EMG	Biochemical genetics	Muscle biopsies	CSF	Prior Genetic Testing (all single gene testing)	Total number of unnecessary previous studies	Number of extra specialized outpatient’s visits	Total estimated expendable cost (USD)	Total diagnostic procedures (non-expendable) (USD)
1	1	2						3	5	2149	2801
2								0	4	1000	2942
3	**1**							1	3	935	3079
4		2						2	6	2214	1957
5	1	2	1				3	7	4	2913	3171
6		1	1					2	5	1792	2730
7	1	2						3	2	1399	3137
8								0	4	1000	3194
9			1					1	6	1614	2564
10		1						1	8	2357	2237
11		1						1	4	1357	2637
12	1	2	1					4	6	2513	1641
13	1	1					1	3	5	842	4941
14		1						1	4	1357	2871
15			1					1	3	814	3678
16								0	4	1000	3478
17		2					1	3	6	2514	3357
18		2					1	3	5	2264	3221
19		1						1	3	1107	3101
20		2					1	3	2	1157	3314
21		1						1	4	1357	2837
22		2						2	6	2214	2757
23								0	5	1250	3214
24								0	4	1000	3364
25	1	2						3	5	2149	2250
26		1						1	6	1857	2457
27			2					2	7	1978	2443
28								0	6	1500	3178
29			2		1			3	3	1585	2928
30		1						1	6	1857	2478
31		1						1	4	1357	2757
32	1	1						2	7	2292	2387
33		1	3					4	6	2199	3000
34		1						1	4	1114	3000
35			1					1	2	614	3278
36		1						2	4	1357	2757
37		2						2	6	2214	2100
38		1						3	4	1828	2951
39	2	1						3	3	1977	2407
40	1	1				1		3	5	1872	3044

* Only repetitive procedures and visits were considered unnecessary. Thus, only them were summed up for the costs of diagnostic odysseys.

## Discussion

Applying WES to a representative sample of 40 patients suffering from neurogenetic diseases, we obtained an etiologic diagnostic yield of 40%. Furthermore, we were able to expand the phenotypic spectrum of known genes and identify new pathogenic variants in other genes. Two cases were illustrative of common themes in medical genomics [17, 18]. A non-sense mutation in *GRIK2* caused a more complex phenotype than it was previously recognized for this gene. This gene encodes a glutamate receptor and was previously reported once in members of a consanguineous family segregating intellectual disability [19]. Our patient also presented with intellectual disability, epilepsy, dystonia, and behavioral problems of the autism spectrum [20]. Thus, we were able to extend the phenotypic spectrum associated with this gene. We also emphasize the finding of a mutation in *KCNA2* in a patient with early onset epilepsy and ataxia. This variant was identified after periodic reanalysis of previously non-diagnostic WES. Mutations in *KCNA2* were recently recognized as the cause of epileptic encephalopathies and early onset ataxia [21]. This information was unknown at the moment of the initial analysis, however, being available when this WES was reassessed, it led us to reinterpret this case. Recent reports have shown that systematic re-analysis of unsolved WES data lead to about 10% additional diagnoses [22].

Our preliminary cost-analysis lend support to the assertion made by others that WES is more cost-effective than other molecular diagnostic approaches based on single- or panel- gene analysis [2, 3]. However, our estimates ought to be interpreted with caution. The retrospective design precludes us to avoid biases during the classification of previous procedures as unnecessary or evitable. We acknowledge that some of them could certainly be useful for WES interpretation and should not be considered a complete cost to be saved by WES. Nevertheless, our findings are similar to other formal analyses in this subject [23], where an early implementation of WES in the diagnostic trajectory of suspected genetic conditions proves to be cost-effective by means of a reduction in the number of procedures and specialist visits [24]. Moreover, there are other *diagnostic odysseys* costs that are harder to represent in monetary terms but are not less important, such as time lost to the patient and family and quality of life decrement because of this loss. They deserve other type of formal economic studies that could even show more advantages for the use of WES in the diagnostic approach of complex diseases such as neurogenetic disorders.

The diagnostic yield in less restrictive adult and pediatric populations series ranged from 17 to 30% [4, 25]. Groups that included only patients showing phenotypes involving the nervous system reported higher diagnostic yields [26–28]. Our results are comparable with these experiences and highlight the advantages of working as a personalized research group where phenotypic and genotypic information can be thoughtfully assessed in contrast to commercial diagnostic laboratories that only have access to focused, heterogeneous and often less informative clinical phenotypic reports filled by the external ordering physician. Although undirected next generation sequencing tests such as WES have proved powerful and useful in the diagnosis of several genetic conditions, a targeted approach based on multi-genic panels or even single-gene assays is still justified for patients presenting with well-defined phenotypes where a higher diagnostic yield might be expected because of better coverage and more favorable cost implications [29]. However, WES have the advantage over more focused approaches, when a more comprehensive solution is needed in those patients suffering from genetically and phenotypically heterogeneous conditions [30, 31].

WES for neurogenetics proved to be an effective, cost- and time-saving approach for the molecular diagnosis of this heterogeneous and complex group of patients. It reduces the long time that these patients must wait before getting a diagnosis thereby ending *odysseys* of many years, impacting on their medical management, and optimizing the genetic counseling for these families. Negative WES still remain a challenge, given the complexity of genomic data interpretation and the lack of a thorough knowledge of monogenic disorders.

## Supporting information

S1 Table Novel variants. (DOCX)

S1 Fig Data analysis and interpretation workflow. (TIFF)

S2 Fig Characteristics and diagnostic yield of our cohort according to phenotype. (TIFF)
