# Supplementary material for: Whole exome sequencing in neurogenetic odysseys: An effective, cost- and time-saving diagnostic approach
Source: PLoS One. 2018 Feb 1;13(2):e0191228. doi: 10.1371/journal.pone.0191228 (PMC5794057; doi:10.1371/journal.pone.0191228)

**S1 Table. Novel Variants**

| case | Variant | ACMG 2015 Criteria | ACMG Classification |
| --- | --- | --- | --- |
| 1 | NM_021956.4:c592C>T; p.Arg198ter | PVS1 | LP |
|  |  | PM2 |  |
| 2 | NM_001242896:c.4718T>C;p.Leu1573Pro | PM2 | LP |
|  |  | PP1 |  |
|  |  | PP2 |  |
|  |  | PP3 |  |
|  |  | PP4 |  |
| 4 | NM_000068: c.3675C>A; p.Phe1225Leu | PM2 | LP |
|  |  | PM6 |  |
|  |  | PP2 |  |
|  |  | PP3 |  |
| 5 | NM_005861.2:c.612+1 G> C; p.? | PVS1 | P |
|  |  | PM2 |  |
|  |  | PP3 |  |
| 5 | NM_005861.2:c.823C>G;Leu275Val | PM2 | LP |
|  |  | PM3 |  |
|  |  | PP2 |  |
|  |  | PP3 |  |
| 6 | NM_025137:c.6763insA;p.Leu2255Hisfsx85 | PVS1 | P |
|  |  | PM2 |  |
|  |  | PP3 |  |
| 6 | NM_025137:6726A>T; p.Gln2242His | PM2 | LP |
|  |  | PM3 |  |
|  |  | PP2 |  |
|  |  | PP3 |  |
|  |  | PP4 |  |
| 21 | NM_001184880:exon1:c.T1151G:p.Val384Gly | PM1 | LP |
|  |  | PM2 |  |
|  |  | PP2 |  |
|  |  | PP3 |  |
| 26 | NM_007055.3:c.3781G>A; p.Glu1261Lys | PM2 | LP |
|  |  | PM3 |  |
|  |  | PP2 |  |
|  |  | PP3 |  |
| 30 | NM_020988:c.709G>A:p.Glu237Lys | PM2 | LP |
|  |  | PM6 |  |
|  |  | PP2 |  |
|  |  | PP3 |  |
| 33 | NM_020919:exon13:c.T2531A:p.Leu844His | PM2 | VUS |
|  |  | PP2 |  |
|  |  | PP3 |  |
| 40 | NM_000053:c.2165T>A:p.Leu722Gln | PS3 | LP |
|  |  | PM2 |  |
|  |  | PP3 |  |
|  |  | PP4 |  |
| 40 | NM_000053:c.3704G>A:p.Gly235Asp | PS3 | LP |
|  |  | PM2 |  |
|  |  | PP3 |  |
|  |  | PP4 |  |


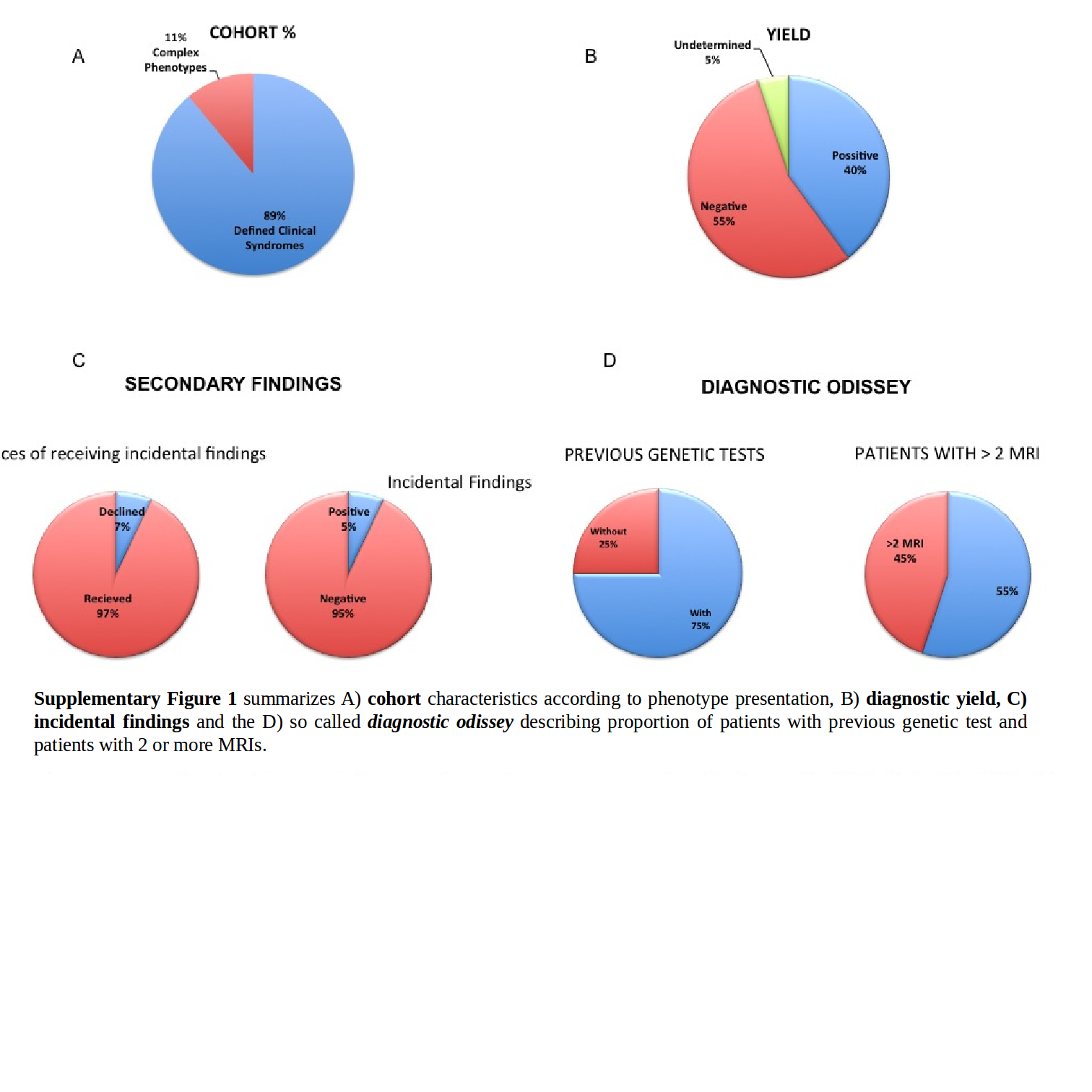


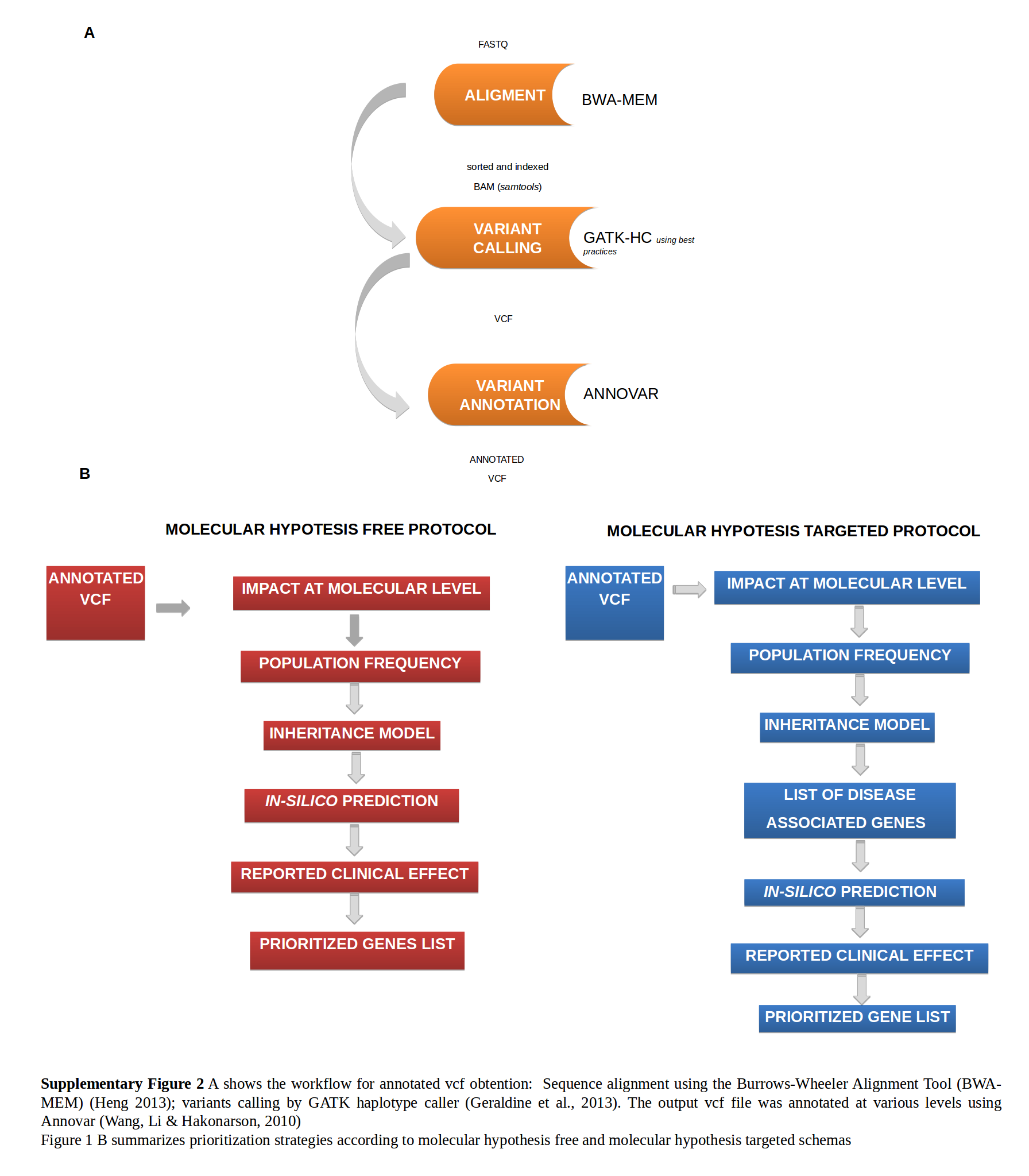

Supplement: S1 Table — (DOCX) [file pone.0191228.s001.docx]
