# Supplementary figures and images for: Whole exome sequencing in neurogenetic odysseys: An effective, cost- and time-saving diagnostic approach
Source: PLoS One. 2018 Feb 1;13(2):e0191228. doi: 10.1371/journal.pone.0191228 (PMC5794057; doi:10.1371/journal.pone.0191228)

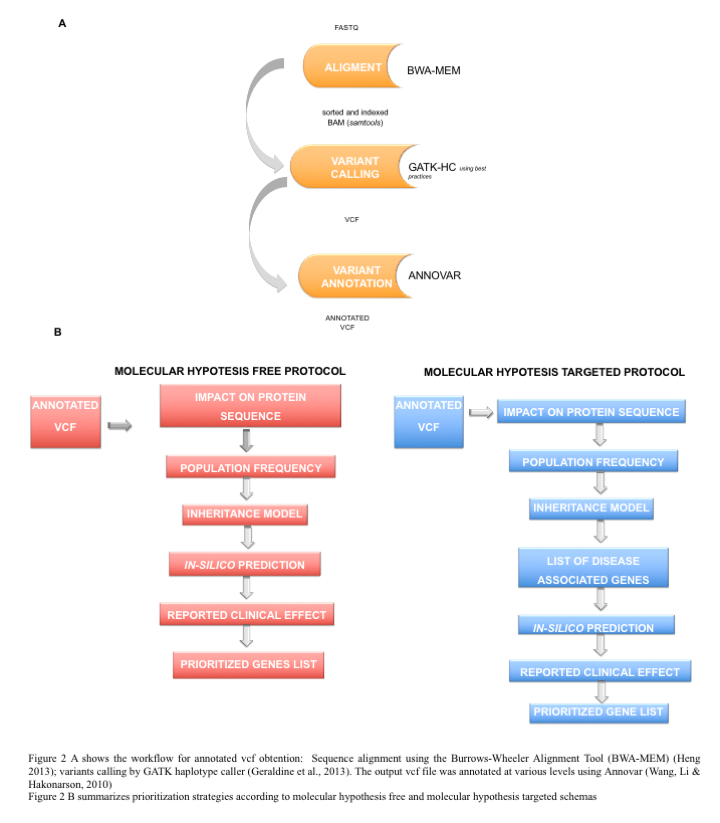

Supplement: S1 Fig — (TIFF) [file pone.0191228.s002.tiff]

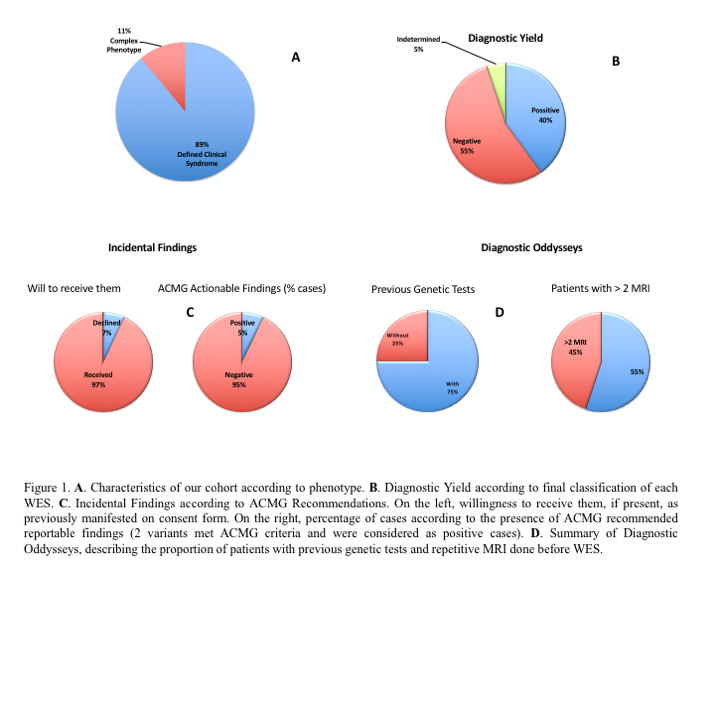

Supplement: S2 Fig — (TIFF) [file pone.0191228.s003.tiff]
